# (Acetone-2κ*O*){μ-6,6′-dimeth­oxy-2,2′-[propane-1,2-diylbis(nitrilo­methyl­idyne)]diphenolato-κ^8^1:2*O* ^6^,*O* ^1^,*O* ^1′^,*O* ^6′^:*O* ^1^,*N*,*N*′,*O* ^1′^}tris­(nitrato-1κ^2^ *O*,*O*′)copper(II)samarium(III)

**DOI:** 10.1107/S160053680902399X

**Published:** 2009-06-27

**Authors:** Wen-Bin Sun, Peng-Fei Yan, Hong-Feng Li, Ting Gao, Guang-Ming Li

**Affiliations:** a Key Laboratory of Functional Inorganic Material Chemistry (HLJU), Ministry of Education, School of Chemistry and Materials Science, Heilongjiang University, Harbin 150080, People’s Republic of China

## Abstract

In the title heteronuclear complex, [CuSm(C_19_H_20_N_2_O_4_)(NO_3_)_3_(CH_3_COCH_3_)], the Cu^II^ ion is five-coordinated by two O and two N atoms from the 6,6′-dimeth­oxy-2,2′-[propane-1,2-diylbis(nitrilo­methyl­idyne)]diphenolate ligand (*L*) and by an O atom from the acetone mol­ecule in a square-pyramidal geometry. The Sm^III^ ion is ten-coordinated by six O atoms from the three nitrate ligands and four O atoms from the *L* ligand. In *L*, the C atoms of the diamino­propane fragment are disordered over two positions in a 0.674 (10):0.326 (10) ratio.

## Related literature

For similar Cu–*Ln* (*Ln* = Gd, Pr and Tb) dinuclear complexes of the *N*,*N*′-bis­(3-methoxy­salicyl­idene)propane-1,2-diamine ligand, see: Kara *et al.* (2000 ▶); Sun *et al.* (2007 ▶, 2009 ▶).
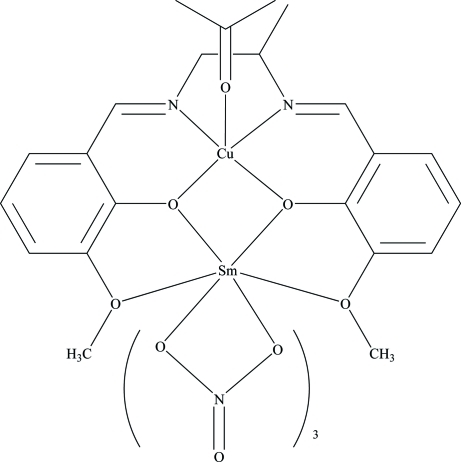


## Experimental

### 

#### Crystal data

[CuSm(C_19_H_20_N_2_O_4_)(NO_3_)_3_(C_3_H_6_O)]
*M* *_r_* = 797.38
Monoclinic, 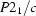

*a* = 9.882 (4) Å
*b* = 18.868 (5) Å
*c* = 15.631 (5) Å
β = 95.320 (16)°
*V* = 2901.9 (16) Å^3^
*Z* = 4
Mo *K*α radiation
μ = 2.81 mm^−1^
*T* = 291 K
0.39 × 0.33 × 0.29 mm

#### Data collection

Rigaku R-AXIS RAPID diffractometer
Absorption correction: multi-scan (*ABSCOR*; Higashi, 1995 ▶) *T*_min_ = 0.404, *T*_max_ = 0.500 (expected range = 0.357–0.442)
28125 measured reflections
6634 independent reflections
5584 reflections with *I* > 2σ(*I*)
*R*_int_ = 0.031

#### Refinement

*R*[*F* ^2^ > 2σ(*F* ^2^)] = 0.033
*wR*(*F* ^2^) = 0.088
*S* = 1.07
6634 reflections
421 parameters
36 restraints
H-atom parameters constrained
Δρ_max_ = 1.03 e Å^−3^
Δρ_min_ = −0.74 e Å^−3^

### 

Data collection: *RAPID-AUTO* (Rigaku, 1998 ▶); cell refinement: *RAPID-AUTO*; data reduction: *CrystalStructure* (Rigaku/MSC, 2002 ▶); program(s) used to solve structure: *SHELXS97* (Sheldrick, 2008 ▶); program(s) used to refine structure: *SHELXL97* (Sheldrick, 2008 ▶); molecular graphics: *SHELXTL* (Sheldrick, 2008 ▶); software used to prepare material for publication: *SHELXL97*.

## Supplementary Material

Crystal structure: contains datablocks I, global. DOI: 10.1107/S160053680902399X/cv2575sup1.cif

Structure factors: contains datablocks I. DOI: 10.1107/S160053680902399X/cv2575Isup2.hkl

Additional supplementary materials:  crystallographic information; 3D view; checkCIF report

## Figures and Tables

**Fig. 1.**
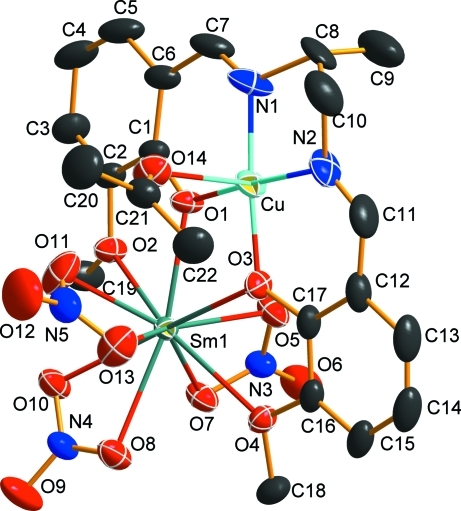
The molecular structure of the title compound showing the atomic numbering and 40% probability displacement ellipsoids. Only major part of the disordered fragment is shown.

## supplementary crystallographic information

### Comment

In continuation of our study of heteronuclear complexes of *N*,*N*'-bis(3- methoxysalicylidene)propane-1,2-diamine ligand (Sun *et al.*, 2007, 2009), we present here the crystal structure of the title compound. As shown in Fig. 1, ligand *L* links Cu and Sm atoms into a dinuclear complex through two phenolate O atoms, and the Sm^III^ centre in the complex is ten-coordinated by four oxygen atoms from *L* and six oxygen atoms from three nitrato ions. The Cu^II^ center is five-coordinate by two nitrogen atoms and two oxygen atoms from the ligand and one oxygen atom from acetone in a square-pyramidal geometry. The title compound is isostructural with the previous Cu—Ln complexes (Ln = Gd, Pr and Tb) (Kara *et al.*, 2000; Sun *et al.*, 2007, 2009) derived from the same ligand.

### Experimental

To a 1:1 MeOH/Me_2_CO solution (20 ml) of the Schiff ligand (0.086 g, 0.250 mmol) was slowly added an aqueous solution (8 ml) of [Cu(Ac)_2_H_2_O] (0.050 g, 0.25 mmol), after refluxing and stirring for 3 h, was slowly added a MeOH solution (10 ml) of Sm(NO_3_)_3_6H_2_O (0.105 g, 0.25 mmol) at ambient temperature. After stirring for 5 h, red solid was collected by filtration and washed with MeOH, [CuSm(C_19_H_20_N_2_O_4_)(CH_3_COCH_3_)(NO_3_)_3_], yield 0.180 g (87%). Single crystals suitable for X-ray determination were obtained by slow diffusion of diethylether into a methanol solution of the powder sample over one week. Analysis calculated for C_22_H_26_CuN_5_O_14_Sm: C, 33.10; H, 3.28; N, 8.77; found: C, 33.01; H, 3.31; N, 8.92%.

### Refinement

H atoms bound to C atoms were placed in calculated positions and treated as riding on their parent atoms, with C—H = 0.93 Å (aromatic C), C—H = 0.98 Å (methylene C), C—H = 0.96 Å (methly C) and with *U*_iso_(H) = 1.2Ueq(C). The C atoms of the diaminopropane fragment were treated as disordered over two positions with the occupancy factors refined to 0.674 (10) and 0.326 (10), respectively.

### Crystal data

**Table:**

[CuSm(C_19_H_20_N_2_O_4_)(NO_3_)_3_(C_3_H_6_O)]	*F*(000) = 1580
*M**_r_* = 797.38	*D*_x_ = 1.825 Mg m^−^^3^
Monoclinic, *P*2_1_/*c*	Mo *K*α radiation, λ = 0.71073 Å
Hall symbol: -P 2ybc	Cell parameters from 23031 reflections
*a* = 9.882 (4) Å	θ = 3.0–27.5°
*b* = 18.868 (5) Å	µ = 2.81 mm^−^^1^
*c* = 15.631 (5) Å	*T* = 291 K
β = 95.320 (16)°	Block, brown
*V* = 2901.9 (16) Å^3^	0.39 × 0.33 × 0.29 mm
*Z* = 4	

### Data collection

**Table:**

Rigaku R-AXIS RAPID diffractometer	6634 independent reflections
Radiation source: fine-focus sealed tube	5584 reflections with *I* > 2σ(*I*)
graphite	*R*_int_ = 0.031
ω scans	θ_max_ = 27.5°, θ_min_ = 3.0°
Absorption correction: multi-scan (*ABSCOR*; Higashi, 1995)	*h* = −12→12
*T*_min_ = 0.404, *T*_max_ = 0.500	*k* = −24→24
28125 measured reflections	*l* = −19→20

### Refinement

**Table:**

Refinement on *F*^2^	Primary atom site location: structure-invariant direct methods
Least-squares matrix: full	Secondary atom site location: difference Fourier map
*R*[*F*^2^ > 2σ(*F*^2^)] = 0.033	Hydrogen site location: inferred from neighbouring sites
*wR*(*F*^2^) = 0.088	H-atom parameters constrained
*S* = 1.07	*w* = 1/[σ^2^(*F*_o_^2^) + (0.0469*P*)^2^ + 1.8845*P*] where *P* = (*F*_o_^2^ + 2*F*_c_^2^)/3
6634 reflections	(Δ/σ)_max_ = 0.006
421 parameters	Δρ_max_ = 1.02 e Å^−^^3^
36 restraints	Δρ_min_ = −0.74 e Å^−^^3^

### Special details

**Table:**

Geometry. All e.s.d.'s (except the e.s.d. in the dihedral angle between two l.s. planes) are estimated using the full covariance matrix. The cell e.s.d.'s are taken into account individually in the estimation of e.s.d.'s in distances, angles and torsion angles; correlations between e.s.d.'s in cell parameters are only used when they are defined by crystal symmetry. An approximate (isotropic) treatment of cell e.s.d.'s is used for estimating e.s.d.'s involving l.s. planes.
Refinement. Refinement of *F*^2^ against ALL reflections. The weighted *R*-factor *wR* and goodness of fit *S* are based on *F*^2^, conventional *R*-factors *R* are based on *F*, with *F* set to zero for negative *F*^2^. The threshold expression of *F*^2^ > σ(*F*^2^) is used only for calculating *R*-factors(gt) *etc*. and is not relevant to the choice of reflections for refinement. *R*-factors based on *F*^2^ are statistically about twice as large as those based on *F*, and *R*- factors based on ALL data will be even larger.

### Fractional atomic coordinates and isotropic or equivalent isotropic displacement parameters (Å^2^)

**Table:**

	*x*	*y*	*z*	*U*_iso_*/*U*_eq_	Occ. (<1)
C8'	0.1263 (19)	0.1131 (8)	0.6707 (10)	0.071 (4)	0.326 (10)
H4'	0.0359	0.1213	0.6897	0.086*	0.326 (10)
C9'	0.145 (3)	0.0470 (10)	0.6393 (18)	0.150 (11)	0.326 (10)
H5A	0.1310	0.0479	0.5778	0.225*	0.326 (10)
H6A	0.0817	0.0149	0.6615	0.225*	0.326 (10)
H7A	0.2362	0.0315	0.6566	0.225*	0.326 (10)
C10'	0.1389 (17)	0.1317 (7)	0.5793 (11)	0.051 (4)	0.326 (10)
H8A	0.0701	0.1072	0.5421	0.061*	0.326 (10)
H9A	0.2277	0.1182	0.5631	0.061*	0.326 (10)
C8	0.1982 (10)	0.1147 (4)	0.6338 (6)	0.075 (2)	0.674 (10)
H4	0.1715	0.0691	0.6569	0.091*	0.674 (10)
C9	0.2955 (13)	0.1009 (7)	0.5751 (7)	0.129 (4)	0.674 (10)
H5	0.3049	0.1418	0.5397	0.193*	0.674 (10)
H6	0.2663	0.0612	0.5396	0.193*	0.674 (10)
H7	0.3815	0.0902	0.6062	0.193*	0.674 (10)
C10	0.0731 (13)	0.1457 (5)	0.5815 (8)	0.096 (4)	0.674 (10)
H8	0.0545	0.1216	0.5268	0.115*	0.674 (10)
H9	−0.0069	0.1437	0.6130	0.115*	0.674 (10)
Sm1	0.28473 (2)	0.426361 (8)	0.751802 (10)	0.03909 (7)	
Cu	0.20496 (6)	0.25917 (2)	0.67412 (3)	0.05590 (14)	
O1	0.3107 (3)	0.30109 (12)	0.76905 (17)	0.0528 (7)	
O2	0.4514 (3)	0.37744 (13)	0.87833 (16)	0.0527 (7)	
O3	0.1956 (3)	0.35392 (13)	0.63401 (16)	0.0525 (7)	
O4	0.2338 (3)	0.48443 (14)	0.59859 (16)	0.0512 (6)	
O5	0.4888 (3)	0.39591 (16)	0.6739 (2)	0.0659 (8)	
O6	0.6496 (5)	0.4695 (2)	0.6546 (3)	0.1085 (16)	
O7	0.4919 (3)	0.49733 (14)	0.7349 (2)	0.0609 (7)	
O8	0.2232 (4)	0.55708 (17)	0.7635 (2)	0.0720 (10)	
O9	0.2694 (5)	0.62299 (18)	0.8738 (3)	0.1104 (17)	
O10	0.3179 (5)	0.51210 (16)	0.87669 (19)	0.0808 (11)	
O11	0.1305 (5)	0.3903 (3)	0.8594 (3)	0.0988 (13)	
O12	−0.0836 (5)	0.4062 (3)	0.8508 (4)	0.132 (2)	
O13	0.0338 (4)	0.4383 (2)	0.7491 (3)	0.0904 (12)	
O14	−0.0249 (4)	0.25774 (19)	0.7448 (2)	0.0749 (9)	
N1	0.2387 (5)	0.16408 (19)	0.7111 (3)	0.0851 (14)	
N2	0.1181 (5)	0.2182 (2)	0.5703 (3)	0.0780 (13)	
N3	0.5473 (4)	0.45422 (18)	0.6863 (2)	0.0566 (8)	
N4	0.2711 (4)	0.56584 (17)	0.8385 (2)	0.0606 (10)	
N5	0.0199 (5)	0.4123 (2)	0.8212 (3)	0.0754 (12)	
C1	0.3693 (4)	0.26602 (18)	0.8360 (3)	0.0465 (8)	
C2	0.4440 (4)	0.30587 (19)	0.8984 (2)	0.0494 (9)	
C3	0.5035 (5)	0.2754 (3)	0.9723 (3)	0.0700 (13)	
H1	0.5525	0.3028	1.0138	0.084*	
C4	0.4889 (6)	0.2021 (3)	0.9838 (4)	0.0815 (16)	
H2	0.5252	0.1809	1.0346	0.098*	
C5	0.4224 (5)	0.1622 (2)	0.9217 (4)	0.0770 (15)	
H3	0.4164	0.1135	0.9298	0.092*	
C6	0.3628 (4)	0.1918 (2)	0.8460 (3)	0.0559 (10)	
C7	0.3007 (5)	0.1443 (2)	0.7817 (4)	0.0759 (15)	
C11	0.0826 (6)	0.2515 (3)	0.5020 (3)	0.0829 (16)	
H10	0.0442	0.2253	0.4555	0.099*	
C12	0.0966 (5)	0.3270 (3)	0.4898 (3)	0.0638 (12)	
C13	0.0536 (5)	0.3555 (4)	0.4084 (3)	0.0800 (16)	
H11	0.0141	0.3258	0.3656	0.096*	
C14	0.0686 (6)	0.4248 (3)	0.3915 (3)	0.0792 (16)	
H12	0.0377	0.4423	0.3375	0.095*	
C15	0.1281 (5)	0.4698 (3)	0.4517 (2)	0.0652 (12)	
H13	0.1398	0.5174	0.4387	0.078*	
C16	0.1710 (4)	0.4441 (2)	0.5324 (2)	0.0508 (9)	
C17	0.1542 (4)	0.3732 (2)	0.5535 (2)	0.0511 (9)	
C18	0.2810 (6)	0.5531 (2)	0.5755 (3)	0.0662 (12)	
H14	0.2046	0.5825	0.5569	0.099*	
H15	0.3388	0.5484	0.5298	0.099*	
H16	0.3311	0.5744	0.6245	0.099*	
C19	0.5576 (6)	0.4172 (2)	0.9257 (3)	0.0725 (14)	
H17	0.5695	0.4616	0.8975	0.109*	
H18	0.6409	0.3907	0.9283	0.109*	
H19	0.5335	0.4257	0.9829	0.109*	
C20	−0.2540 (7)	0.2557 (4)	0.7781 (5)	0.108 (2)	
H20	−0.3030	0.2983	0.7886	0.162*	
H21	−0.2161	0.2362	0.8319	0.162*	
H22	−0.3149	0.2218	0.7493	0.162*	
C21	−0.1422 (5)	0.2722 (2)	0.7232 (3)	0.0635 (11)	
C22	−0.1820 (6)	0.3064 (3)	0.6394 (4)	0.0856 (16)	
H23	−0.2482	0.3426	0.6467	0.128*	
H24	−0.2203	0.2715	0.5995	0.128*	
H25	−0.1035	0.3272	0.6177	0.128*	

### Atomic displacement parameters (Å^2^)

**Table:**

	*U*^11^	*U*^22^	*U*^33^	*U*^12^	*U*^13^	*U*^23^
C8'	0.072 (5)	0.071 (5)	0.072 (5)	−0.0003 (10)	0.0066 (11)	−0.0003 (10)
C9'	0.158 (14)	0.134 (13)	0.159 (14)	−0.015 (9)	0.018 (9)	0.004 (9)
C10'	0.056 (7)	0.029 (5)	0.069 (7)	−0.006 (5)	0.015 (6)	−0.023 (5)
C8	0.077 (2)	0.073 (2)	0.076 (2)	−0.0005 (10)	0.0069 (10)	−0.0024 (10)
C9	0.129 (5)	0.128 (4)	0.129 (4)	0.0003 (10)	0.0126 (11)	−0.0001 (10)
C10	0.101 (9)	0.075 (6)	0.107 (7)	0.005 (6)	−0.011 (7)	−0.049 (5)
Sm1	0.04727 (12)	0.03289 (10)	0.03616 (10)	−0.00080 (7)	−0.00128 (7)	−0.00168 (6)
Cu	0.0668 (3)	0.0395 (2)	0.0598 (3)	−0.0081 (2)	−0.0022 (2)	−0.0138 (2)
O1	0.0649 (19)	0.0321 (11)	0.0580 (15)	−0.0026 (11)	−0.0123 (13)	0.0002 (10)
O2	0.0618 (18)	0.0416 (12)	0.0507 (14)	−0.0020 (12)	−0.0166 (13)	0.0029 (11)
O3	0.0677 (19)	0.0485 (14)	0.0396 (13)	−0.0129 (13)	−0.0046 (12)	−0.0065 (10)
O4	0.0583 (17)	0.0523 (14)	0.0421 (13)	−0.0003 (12)	−0.0003 (12)	0.0079 (11)
O5	0.065 (2)	0.0495 (15)	0.085 (2)	−0.0083 (14)	0.0213 (17)	−0.0209 (15)
O6	0.092 (3)	0.073 (2)	0.172 (4)	−0.019 (2)	0.074 (3)	−0.014 (3)
O7	0.0613 (19)	0.0437 (14)	0.0786 (19)	−0.0055 (13)	0.0112 (15)	−0.0144 (13)
O8	0.099 (3)	0.0538 (16)	0.0581 (17)	0.0220 (17)	−0.0187 (17)	−0.0100 (13)
O9	0.175 (5)	0.0548 (19)	0.092 (3)	0.032 (2)	−0.038 (3)	−0.0323 (18)
O10	0.134 (3)	0.0544 (17)	0.0495 (16)	0.0307 (19)	−0.0160 (18)	−0.0106 (13)
O11	0.080 (3)	0.139 (4)	0.081 (3)	0.004 (3)	0.028 (2)	0.038 (3)
O12	0.087 (3)	0.167 (5)	0.151 (5)	0.000 (3)	0.069 (4)	−0.007 (4)
O13	0.061 (2)	0.117 (3)	0.093 (3)	0.005 (2)	0.008 (2)	0.022 (2)
O14	0.060 (2)	0.082 (2)	0.082 (2)	0.0122 (17)	0.0029 (18)	0.0157 (17)
N1	0.091 (3)	0.0357 (18)	0.124 (4)	−0.0006 (19)	−0.016 (3)	−0.015 (2)
N2	0.093 (3)	0.071 (2)	0.070 (3)	−0.030 (2)	0.010 (2)	−0.036 (2)
N3	0.056 (2)	0.0450 (17)	0.070 (2)	−0.0027 (16)	0.0151 (18)	0.0000 (16)
N4	0.078 (3)	0.0443 (17)	0.057 (2)	0.0107 (16)	−0.0078 (19)	−0.0121 (14)
N5	0.070 (3)	0.074 (3)	0.087 (3)	−0.008 (2)	0.032 (3)	−0.007 (2)
C1	0.043 (2)	0.0379 (17)	0.059 (2)	0.0019 (14)	0.0029 (17)	0.0066 (15)
C2	0.044 (2)	0.0475 (19)	0.055 (2)	0.0063 (16)	−0.0028 (17)	0.0117 (16)
C3	0.067 (3)	0.068 (3)	0.071 (3)	0.003 (2)	−0.016 (2)	0.015 (2)
C4	0.072 (3)	0.074 (3)	0.095 (4)	0.007 (3)	−0.011 (3)	0.044 (3)
C5	0.054 (3)	0.050 (2)	0.126 (5)	0.004 (2)	0.001 (3)	0.033 (3)
C6	0.041 (2)	0.0401 (18)	0.086 (3)	0.0040 (16)	0.004 (2)	0.0095 (18)
C7	0.062 (3)	0.0345 (19)	0.129 (5)	0.0030 (19)	−0.003 (3)	0.000 (2)
C11	0.087 (4)	0.105 (4)	0.056 (3)	−0.030 (3)	0.004 (3)	−0.038 (3)
C12	0.054 (3)	0.092 (3)	0.045 (2)	−0.011 (2)	0.0046 (18)	−0.021 (2)
C13	0.062 (3)	0.133 (5)	0.042 (2)	−0.006 (3)	−0.007 (2)	−0.023 (3)
C14	0.060 (3)	0.135 (5)	0.041 (2)	0.010 (3)	−0.005 (2)	0.000 (3)
C15	0.054 (3)	0.100 (3)	0.042 (2)	0.015 (2)	0.0065 (19)	0.009 (2)
C16	0.045 (2)	0.072 (2)	0.0357 (17)	0.0054 (18)	0.0040 (15)	0.0003 (16)
C17	0.047 (2)	0.071 (2)	0.0347 (17)	−0.0033 (18)	0.0016 (15)	−0.0089 (16)
C18	0.077 (3)	0.059 (2)	0.063 (3)	−0.004 (2)	0.009 (2)	0.019 (2)
C19	0.079 (3)	0.056 (2)	0.075 (3)	−0.011 (2)	−0.033 (3)	−0.001 (2)
C20	0.077 (4)	0.131 (6)	0.122 (5)	−0.005 (4)	0.033 (4)	0.010 (5)
C21	0.055 (3)	0.058 (2)	0.078 (3)	0.003 (2)	0.006 (2)	−0.001 (2)
C22	0.065 (3)	0.092 (4)	0.097 (4)	0.017 (3)	−0.005 (3)	0.012 (3)

### Geometric parameters (Å, °)

**Table:**

C8'—C9'	1.358 (10)	O8—N4	1.234 (4)
C8'—C10'	1.49 (2)	O9—N4	1.212 (4)
C8'—N1	1.559 (17)	O10—N4	1.243 (4)
C8'—H4'	0.9800	O11—N5	1.266 (6)
C9'—H5A	0.9600	O12—N5	1.166 (5)
C9'—H6A	0.9600	O13—N5	1.249 (6)
C9'—H7A	0.9600	O14—C21	1.208 (6)
C10'—N2	1.648 (15)	N1—C7	1.267 (7)
C10'—H8A	0.9700	N2—C11	1.261 (7)
C10'—H9A	0.9700	C1—C2	1.388 (5)
C8—C9	1.414 (9)	C1—C6	1.412 (5)
C8—C10	1.533 (15)	C2—C3	1.373 (5)
C8—N1	1.549 (9)	C3—C4	1.405 (7)
C8—H4	0.9800	C3—H1	0.9300
C9—H5	0.9600	C4—C5	1.350 (8)
C9—H6	0.9600	C4—H2	0.9300
C9—H7	0.9600	C5—C6	1.389 (6)
C10—N2	1.455 (12)	C5—H3	0.9300
C10—H8	0.9700	C6—C7	1.441 (7)
C10—H9	0.9700	C11—C12	1.444 (8)
Sm1—O1	2.390 (2)	C11—H10	0.9300
Sm1—O3	2.394 (2)	C12—C17	1.404 (5)
Sm1—O11	2.467 (4)	C12—C13	1.410 (7)
Sm1—O7	2.481 (3)	C13—C14	1.346 (8)
Sm1—O13	2.486 (4)	C13—H11	0.9300
Sm1—O5	2.517 (3)	C14—C15	1.361 (7)
Sm1—O10	2.533 (3)	C14—H12	0.9300
Sm1—O8	2.551 (3)	C15—C16	1.380 (5)
Sm1—O2	2.621 (3)	C15—H13	0.9300
Sm1—O4	2.639 (3)	C16—C17	1.392 (6)
Sm1—Cu	3.4452 (9)	C18—H14	0.9600
Cu—O3	1.894 (3)	C18—H15	0.9600
Cu—N1	1.905 (4)	C18—H16	0.9600
Cu—O1	1.906 (3)	C19—H17	0.9600
Cu—N2	1.926 (4)	C19—H18	0.9600
Cu—O14	2.616 (4)	C19—H19	0.9600
O1—C1	1.325 (4)	C20—C21	1.495 (8)
O2—C2	1.390 (4)	C20—H20	0.9600
O2—C19	1.438 (5)	C20—H21	0.9600
O3—C17	1.337 (4)	C20—H22	0.9600
O4—C16	1.384 (5)	C21—C22	1.479 (7)
O4—C18	1.435 (5)	C22—H23	0.9600
O5—N3	1.250 (5)	C22—H24	0.9600
O6—N3	1.202 (5)	C22—H25	0.9600
O7—N3	1.271 (4)		
			
C9'—C8'—C10'	81.1 (16)	C2—O2—Sm1	118.0 (2)
C9'—C8'—N1	126.6 (19)	C19—O2—Sm1	125.6 (2)
C10'—C8'—N1	97.1 (12)	C17—O3—Cu	124.7 (2)
C9'—C8'—H4'	114.5	C17—O3—Sm1	128.8 (2)
C10'—C8'—H4'	114.5	Cu—O3—Sm1	106.33 (11)
N1—C8'—H4'	114.5	C16—O4—C18	116.3 (3)
C8'—C9'—H5A	109.4	C16—O4—Sm1	119.0 (2)
C8'—C9'—H6A	109.4	C18—O4—Sm1	124.3 (2)
H5A—C9'—H6A	109.5	N3—O5—Sm1	96.0 (2)
C8'—C9'—H7A	109.6	N3—O7—Sm1	97.1 (2)
H5A—C9'—H7A	109.5	N4—O8—Sm1	97.2 (2)
H6A—C9'—H7A	109.5	N4—O10—Sm1	97.8 (2)
C8'—C10'—N2	107.2 (10)	N5—O11—Sm1	98.6 (3)
C8'—C10'—H8A	110.3	N5—O13—Sm1	98.2 (3)
N2—C10'—H8A	110.3	C21—O14—Cu	136.6 (3)
C8'—C10'—H9A	110.3	C7—N1—C8	124.8 (5)
N2—C10'—H9A	110.3	C7—N1—C8'	116.1 (7)
H8A—C10'—H9A	108.5	C8—N1—C8'	35.8 (6)
C9—C8—C10	106.7 (10)	C7—N1—Cu	126.8 (3)
C9—C8—N1	118.4 (8)	C8—N1—Cu	107.7 (4)
C10—C8—N1	109.0 (6)	C8'—N1—Cu	111.1 (6)
C9—C8—H4	107.4	C11—N2—C10	120.5 (6)
C10—C8—H4	107.4	C11—N2—C10'	126.1 (7)
N1—C8—H4	107.4	C10—N2—C10'	25.3 (6)
N2—C10—C8	100.5 (8)	C11—N2—Cu	125.5 (3)
N2—C10—H8	111.7	C10—N2—Cu	113.2 (5)
C8—C10—H8	111.7	C10'—N2—Cu	106.4 (7)
N2—C10—H9	111.7	O6—N3—O5	122.8 (4)
C8—C10—H9	111.7	O6—N3—O7	121.2 (4)
H8—C10—H9	109.4	O5—N3—O7	115.9 (3)
O1—Sm1—O3	63.26 (9)	O9—N4—O8	122.1 (4)
O1—Sm1—O11	73.56 (14)	O9—N4—O10	121.8 (4)
O3—Sm1—O11	99.22 (14)	O8—N4—O10	116.1 (3)
O1—Sm1—O7	117.70 (10)	O12—N5—O13	124.8 (6)
O3—Sm1—O7	118.33 (10)	O12—N5—O11	122.1 (6)
O11—Sm1—O7	142.13 (14)	O13—N5—O11	113.1 (4)
O1—Sm1—O13	100.85 (12)	O1—C1—C2	116.7 (3)
O3—Sm1—O13	75.16 (13)	O1—C1—C6	124.2 (4)
O11—Sm1—O13	50.10 (14)	C2—C1—C6	119.1 (4)
O7—Sm1—O13	141.33 (11)	C3—C2—C1	121.5 (4)
O1—Sm1—O5	75.31 (10)	C3—C2—O2	124.7 (4)
O3—Sm1—O5	75.62 (10)	C1—C2—O2	113.8 (3)
O11—Sm1—O5	147.14 (14)	C2—C3—C4	118.7 (5)
O7—Sm1—O5	50.62 (9)	C2—C3—H1	120.7
O13—Sm1—O5	148.77 (13)	C4—C3—H1	120.7
O1—Sm1—O10	122.67 (9)	C5—C4—C3	120.4 (4)
O3—Sm1—O10	165.75 (12)	C5—C4—H2	119.8
O11—Sm1—O10	72.23 (16)	C3—C4—H2	119.8
O7—Sm1—O10	71.85 (12)	C4—C5—C6	121.9 (4)
O13—Sm1—O10	90.71 (15)	C4—C5—H3	119.1
O5—Sm1—O10	117.81 (13)	C6—C5—H3	119.1
O1—Sm1—O8	166.37 (12)	C5—C6—C1	118.3 (4)
O3—Sm1—O8	122.28 (10)	C5—C6—C7	117.6 (4)
O11—Sm1—O8	92.93 (15)	C1—C6—C7	124.1 (4)
O7—Sm1—O8	71.98 (12)	N1—C7—C6	124.4 (4)
O13—Sm1—O8	70.68 (14)	N2—C11—C12	125.5 (4)
O5—Sm1—O8	117.56 (12)	N2—C11—H10	117.2
O10—Sm1—O8	48.83 (10)	C12—C11—H10	117.2
O1—Sm1—O2	60.88 (8)	C17—C12—C13	118.1 (5)
O3—Sm1—O2	122.66 (9)	C17—C12—C11	123.8 (4)
O11—Sm1—O2	76.89 (13)	C13—C12—C11	118.1 (4)
O7—Sm1—O2	78.67 (10)	C14—C13—C12	121.2 (5)
O13—Sm1—O2	126.90 (12)	C14—C13—H11	119.4
O5—Sm1—O2	79.01 (11)	C12—C13—H11	119.4
O10—Sm1—O2	67.43 (9)	C13—C14—C15	121.3 (5)
O8—Sm1—O2	115.05 (9)	C13—C14—H12	119.4
O1—Sm1—O4	121.49 (9)	C15—C14—H12	119.4
O3—Sm1—O4	60.90 (9)	C14—C15—C16	119.3 (5)
O11—Sm1—O4	130.92 (13)	C14—C15—H13	120.3
O7—Sm1—O4	76.65 (10)	C16—C15—H13	120.3
O13—Sm1—O4	80.85 (12)	C15—C16—O4	124.6 (4)
O5—Sm1—O4	75.50 (11)	C15—C16—C17	121.4 (4)
O10—Sm1—O4	115.74 (9)	O4—C16—C17	114.0 (3)
O8—Sm1—O4	68.73 (9)	O3—C17—C16	116.8 (3)
O2—Sm1—O4	152.18 (10)	O3—C17—C12	124.6 (4)
O1—Sm1—Cu	32.10 (6)	C16—C17—C12	118.6 (4)
O3—Sm1—Cu	31.84 (6)	O4—C18—H14	109.5
O11—Sm1—Cu	81.50 (12)	O4—C18—H15	109.5
O7—Sm1—Cu	128.37 (6)	H14—C18—H15	109.5
O13—Sm1—Cu	83.23 (11)	O4—C18—H16	109.5
O5—Sm1—Cu	77.75 (7)	H14—C18—H16	109.5
O10—Sm1—Cu	149.93 (8)	H15—C18—H16	109.5
O8—Sm1—Cu	149.58 (8)	O2—C19—H17	109.5
O2—Sm1—Cu	92.91 (6)	O2—C19—H18	109.5
O4—Sm1—Cu	92.42 (6)	H17—C19—H18	109.5
O3—Cu—N1	172.44 (19)	O2—C19—H19	109.5
O3—Cu—O1	82.66 (11)	H17—C19—H19	109.5
N1—Cu—O1	94.96 (16)	H18—C19—H19	109.5
O3—Cu—N2	95.50 (16)	C21—C20—H20	109.5
N1—Cu—N2	86.0 (2)	C21—C20—H21	109.5
O1—Cu—N2	172.76 (17)	H20—C20—H21	109.5
O3—Cu—O14	97.58 (12)	C21—C20—H22	109.5
N1—Cu—O14	89.80 (18)	H20—C20—H22	109.5
O1—Cu—O14	96.32 (13)	H21—C20—H22	109.5
N2—Cu—O14	90.86 (17)	O14—C21—C22	121.1 (5)
O3—Cu—Sm1	41.83 (7)	O14—C21—C20	122.2 (5)
N1—Cu—Sm1	136.67 (14)	C22—C21—C20	116.7 (5)
O1—Cu—Sm1	41.80 (7)	C21—C22—H23	109.5
N2—Cu—Sm1	137.23 (14)	C21—C22—H24	109.5
O14—Cu—Sm1	92.28 (8)	H23—C22—H24	109.5
C1—O1—Cu	125.1 (2)	C21—C22—H25	109.5
C1—O1—Sm1	128.1 (2)	H23—C22—H25	109.5
Cu—O1—Sm1	106.11 (11)	H24—C22—H25	109.5
C2—O2—C19	116.2 (3)		
